# Repurposing an anti‐cancer agent for the treatment of hypertrophic heart disease

**DOI:** 10.1002/path.5340

**Published:** 2019-10-30

**Authors:** Matthew Dukinfield, Eleni Maniati, Louise E Reynolds, Aisah Aubdool, Reshma S Baliga, Gabriela D'Amico, Oscar Maiques, Jun Wang, Kenneth C Bedi, Kenneth B Margulies, Victoria Sanz‐Moreno, Adrian Hobbs, Kairbaan Hodivala‐Dilke

**Affiliations:** ^1^ Barts Cancer Institute, Queen Mary University of London, John Vane Science Centre, Charterhouse Square London UK; ^2^ William Harvey Research Institute, Queen Mary University of London, Charterhouse Square London UK; ^3^ Perelman School of Medicine University of Pennsylvania, Translational Research Center Philadelphia PA USA

**Keywords:** heart failure, blood vessels, hypertrophy, integrins, angiogenesis, cardiomyocyte, ischaemia, cilengitide

## Abstract

Coronary microvascular dysfunction combined with maladaptive cardiomyocyte morphology and energetics is a major contributor to heart failure advancement. Thus, dually enhancing cardiac angiogenesis and targeting cardiomyocyte function to slow, or reverse, the development of heart failure is a logical step towards improved therapy. We present evidence for the potential to repurpose a former anti‐cancer Arg‐Gly‐Asp (RGD)‐mimetic pentapeptide, cilengitide, here used at low doses. Cilengitide targets αvβ3 integrin and this protein is upregulated in human dilated and ischaemic cardiomyopathies. Treatment of mice after abdominal aortic constriction (AAC) surgery with low‐dose cilengitide (ldCil) enhances coronary angiogenesis and directly affects cardiomyocyte hypertrophy with an associated reduction in disease severity. At a molecular level, ldCil treatment has a direct effect on cardiac endothelial cell transcriptomic profiles, with a significant enhancement of pro‐angiogenic signalling pathways, corroborating the enhanced angiogenic phenotype after ldCil treatment. Moreover, ldCil treatment of Angiotensin II‐stimulated AngII‐stimulated cardiomyocytes significantly restores transcriptomic profiles similar to those found in normal human heart. The significance of this finding is enhanced by transcriptional similarities between AngII‐treated cardiomyocytes and failing human hearts. Taken together, our data provide evidence supporting a possible new strategy for improved heart failure treatment using low‐dose RGD‐mimetics with relevance to human disease. © 2019 The Authors. *The Journal of Pathology* published by John Wiley & Sons Ltd on behalf of Pathological Society of Great Britain and Ireland.

## Introduction

Heart failure affects approximately 26 million people worldwide and is characterised by insufficient cardiac function, poor coronary microvascular reactivity, and maladaptive cardiomyocyte hypertrophy. Despite significant therapeutic advances, none of the current treatment strategies provides long‐term benefit since the disease is associated with a 5‐year mortality of almost 50% 1, 2, 3, 4, 5, 6, 7, 8, 9, 10, 11. Given also that current standard‐of‐care treatments, such as β‐blockers or diuretics, are generally administered indefinitely, can cause serious side effects, and do not fully mitigate heart failure‐related morbidity and mortality, alternative, disease‐modulating solutions are still required.

Integrins are heterodimeric transmembrane adhesion molecules composed of one α‐subunit and one β‐subunit 12. Apart from their roles in adhesion, integrins have also been shown to be allosteric, thus affecting their signalling capacity. The clinical application of inhibiting integrin adhesive functions *in vivo* has proved to be effective (i.e. the anti‐platelet effects of αIIβ3 integrin antagonists), but targeting the vitronectin receptor, αvβ3 integrin 13, 14, appears to be less successful. More recently, however, methods to target the signalling functions of integrins separate from their adhesive roles have emerged, especially for αvβ3 integrin. Interestingly, whilst αvβ3 integrin is expressed at low levels in the normal heart, it is upregulated in endothelial cells and cardiomyocytes of diseased heart 15, 16. Indeed, vascular αvβ3‐targeting probes have been used to image angiogenic areas in damaged heart tissue because αvβ3 is upregulated in angiogenic blood vessels 17, 18, 19, 20. These studies and others have also revealed elevated endothelial αvβ3 expression after myocardial infarction 17, 21. However, targeting αvβ3 signalling without affecting its adhesive function to control hypertrophic heart disease has not been reported previously.

Cilengitide was developed originally as an anti‐angiogenic/anti‐cancer cyclic RGD‐mimetic antagonist of αvβ3 integrin when used at maximally tolerated doses (5–50 mg/kg) 22, 23, 24, 25, 26. In contrast to its antagonistic anti‐adhesive and anti‐angiogenic effects at these doses, we published that low doses of cilengitide (ldCil, 50 μg/kg *in vivo* or 2 nm *in vitro*) enhanced pathological angiogenesis with no apparent effects on quiescent vasculature. Mechanistically, ldCil does not act as an αvβ3‐integrin antagonist and accordingly does not affect cell adhesion or migration, but instead alters αvβ3 signalling to enhance the activation and recycling of the major pro‐angiogenic receptor VEGF‐receptor 2 27. We have exploited these features of ldCil to improve chemotherapy efficacy in cancer models *in vivo* by actually increasing tumour blood vessel number and directly enhancing chemotherapy delivery and metabolism in malignant cells 28. Taken together, these data support ldCil treatment as a strategy to affect αvβ3‐integrin signalling without affecting cell adhesion and migration.

Here we provide evidence to rationalise the repurposing of ldCil for the treatment of heart failure. We show that treatment with ldCil in abdominal aortic constriction, a well‐established mouse model of heart failure, restores cardiac function to near‐normal levels. Indeed, we provide evidence that ldCil treatment enhances cardiac angiogenesis with a correlative increase in endothelial cell activation at the transcriptomic level. In complementary studies involving comparisons with published Illumina and RNA‐Seq data from normal and human heart failure, we show that angiotensin II (AngII) treatment of mouse cardiomyocytes induces many of the transcriptomic changes observed in human heart failure, and that treatment of AngII‐exposed cardiomyocytes with ldCil restores these transcriptomic changes back towards those found in normal human heart transcriptomic profiles. Together, these data indicate that repurposing cilengitide may have salutary impact on human heart failure.

## Materials and methods

### Human heart tissue

Human myocardial tissue was obtained under protocol ethical regulations approved by Institutional Review Boards at the University of Pennsylvania and the Gift‐of‐Life Donor Program (Pennsylvania, USA). Whole hearts and dissected left ventricle cavity were weighed to determine levels of hypertrophy. Transmural myocardial samples were dissected from the mid‐left ventricular free wall. Snap‐frozen tissue samples and formalin‐fixed, paraffin‐embedded (FFPE) sections were provided. Additional details are provided in supplementary material, Supplementary materials and methods.

### Western blotting analysis of human heart tissue

Less than 100 mg of snap‐frozen tissue was lysed in RIPA buffer supplemented with protease inhibitor cocktail (Merck‐Sigma, Gillingham, UK). Tissue was homogenised using a Polytron tissue homogeniser for 30 s followed by centrifugation, for 15 min at 4 °C, to pellet tissue debris. Lysates were subjected to SDS‐PAGE and transferred to nitrocellulose membranes (Amersham Biosciences, Amersham, UK) for western blotting. Blots were probed for β3 integrin (antibody a kind gift from Barry Coller, Rockefeller University; 1:500), succinate dehydrogenase (ab178423, 1:1000; Abcam, Cambridge, UK), pyruvate dehydrogenase (ab131263, 1:1000; Abcam), aconitase (ab129069, 1:10 000; Abcam), and GAPDH (AB2302; Merck, Hoddeston, UK). Densitometric readings of band intensities were obtained using ImageJ software (NIH, USA).

### Immunofluorescence for β3 integrin in human heart tissue

Paraffin‐embedded tissues were dewaxed, blocked in 10% normal goat serum (NGS) and 1% bovine serum albumin (BSA) for 1 h, followed by incubation with anti‐β3 primary antibody (1:200, clone 2C9.G3, 14‐0611‐0685; Fisher Scientific, Loughborough, UK) in blocking buffer (10% NGS, 1% BSA) overnight. Next, sections were incubated for 2 h at room temperature with secondary antibody in blocking buffer (10% NGS, 1% BSA) conjugated to Alexa Fluor® 488 (1:100; Invitrogen, Loughborough, UK), before being immersed in Sudan Black for 15 min to reduce autofluorescence. Sections were washed and mounted in ProLong Gold with DAPI (Invitrogen). Stained sections were imaged on the Zeiss 710 confocal microscope and processed using ImageJ software.

### Studies in mice

All procedures were approved by the Animal Welfare and Ethical Review Board (AWERB) at Queen Mary University of London and were executed in accordance with UK Home Office Animals (Scientific Procedures) Act 1986.

### Abdominal aortic constriction (AAC)

AAC is a common experimental procedure used to elicit pressure overload‐induced heart failure (HF) in murine models. AAC provides a time‐dependent, reproducible model of HF characterised by a significant rise in left ventricular weight, deleterious myocardial dilation and a reduction in cardiac inotropy, and rise in myocardial fibrosis. Physiologically, this mimics some of the clinical features of human aortic stenosis. In an effort to ensure reproducibility amongst AAC surgeries, only male mice weighing between 20 and 22 g (correlating to 4–5 weeks of age) (Charles River, Harlow, UK) were used. Additional details are provided in supplementary material, Supplementary materials and methods.

### Cilengitide administration

Cilengitide (Bachem, Bubendorf, Switzerland) was stored as a 2 m solution in saline at −20 °C prior to use, before thawing and serial diluting to an appropriate concentration with sterile saline for use. Injections were administered three times per week at the indicated doses intraperitoneally (i.p.). Low‐dose cilengitide (ldCil) is classified as 50 μg/kg 27.

### Echocardiography

Cardiac function was assessed by transthoracic echocardiography using a VisualSonics Vevo 770 30‐MHz transduction probe (VisualSonics, Amsterdam, The Netherlands). Additional details are provided in supplementary material, Supplementary materials and methods.

### Mean arterial blood pressure (MABP)

Blood pressure was recorded in anaesthetised mice via intra‐arterial cannulation of a carotid artery 6 weeks post‐AAC surgery, using a Microtip pressure catheter (Millar Instruments, Oxford, UK). MABP was then averaged across a 30 s period using MATLAB analysis. Additional details are provided in supplementary material, Supplementary materials and methods.

### Immunofluorescence for wheat germ agglutinin (WGA) and CD31 and measurement of cardiomyocyte size and blood vessel density

Immunofluorescence for WGA (W11261; Invitrogen) and endomucin (sc‐65 495, clone V7C7; Santa Cruz, Insight Biotechnology Ltd, Wembley, UK) was performed on 5 μm snap‐frozen heart sections. Additional details are provided in supplementary material, Supplementary materials and methods.

### Cardiac endothelial cell isolation

Primary mouse endothelial cells were isolated from the whole hearts of 3‐ to 7‐day‐old C57/BL6 mice as described previously 29.

### Cardiomyocyte cell isolation

Primary mouse cardiomyocytes were isolated from the whole hearts of 1‐ to 3‐day‐old C57/BL6 wild‐type mice. Additional details are provided in supplementary material, Supplementary materials and methods.

### Western blot analysis of cardiomyocytes

Cardiomyocytes were prepared and treated as above. Cells were lysed with RIPA buffer and 20 μg of protein was loaded onto 10% gels. Membranes were probed for succinate dehydrogenase (ab178423; Abcam), pyruvate dehydrogenase (ab131263; Abcam), aconitase (ab129069; Abcam), and GAPDH (AB2302, 1:5000; Millipore). Densitometric readings of band intensities were obtained using ImageJ software.

### RNA analysis by RT‐qPCR and RNA‐Seq

RNA was extracted from cells using the RNeasy mini kit according to the manufacturer's instructions ( Qiagen UK, Manchester, UK). For whole hearts, less than 100 mg of tissue was lysed with RLT lysis buffer ( Qiagen) in a QIAshredder (Qiagen) for 10 min. Tissues were processed with the RNeasy mini kit. Complementary DNA (cDNA) synthesis was carried out using the Applied Biosystems High Capacity cDNA Reverse Transcription (RT) Kit (Thermo Fisher Scientific Paisley, UK) according to the manufacturer's instructions; 600 ng of mouse tissue/cells cDNA and 1000 ng of human heart tissue cDNA were used for qPCR using sample cDNA (FAM‐tagged), an internal control *Gapdh* (VIC‐tagged), and specific TaqMan probes. qPCR was carried out using the TaqMan Universal PCR Master Mix (PE Applied Biosystems, Thermo Fisher Scientific) in a 96‐well plate. 160 ng of cDNA from each sample was amplified using qPCR across 40 cycles. Target mRNA was normalised to *Gapdh* (*HPRT1* if human), and the expression level of each gene was determined relative to the initial experimental controls using the 2^−ΔΔCT^ method.

### RNA‐Seq analyses and comparison with human heart data

RNA‐Seq was performed by Barts and the London Genome Centre on the Illumina NextSeq 500 platform. Additional details are provided in supplementary material, Supplementary materials and methods.

## Results

### β3‐integrin expression is elevated in human dilated and ischaemic cardiomyopathies

**Figure 1:**
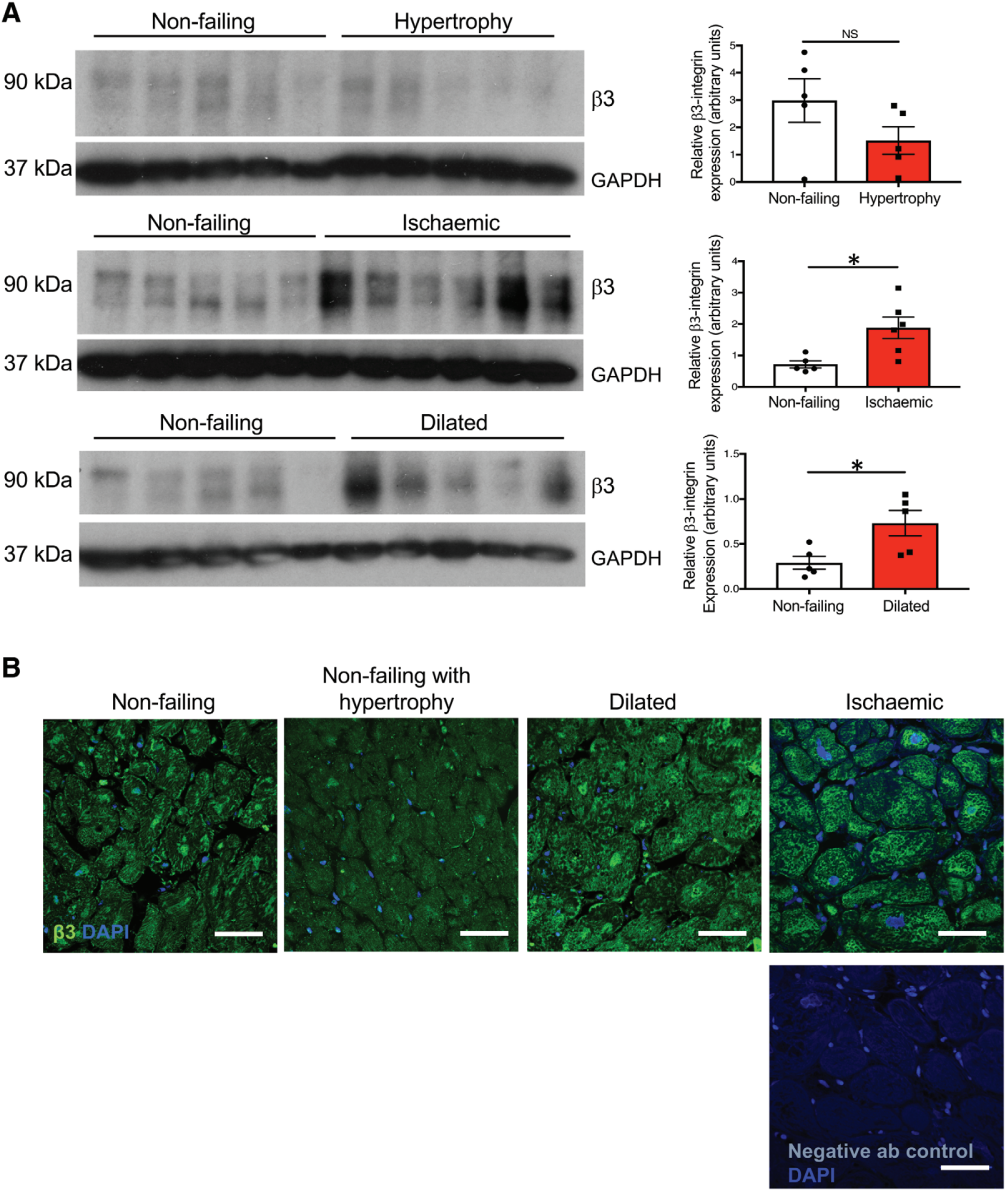
β3‐integrin expression is upregulated in human dilated and ischaemic cardiomyopathy. (A) Western blotting analysis of β3 integrin in protein lysates from human non‐failing heart, non‐failing heart with hypertrophy, and dilated and ischaemic myocardium. GAPDH was used as a loading control. Bar charts show mean densitometric readings ± SEM. *N*, 5 or 6 individual patient samples. (B) Immunofluorescence reveals β3‐integrin expression in sections of human non‐failing heart with an apparent increase in dilated and ischaemic hearts, but less in non‐failing heart with hypertrophy. The non‐specific primary antibody control showed minimal background level staining. *N*, four individual patient samples per group. Scale bar = 50 μm. Student's *t*‐test, *p < 0.05.

To test the utility of low‐dose cilengitide (ldCil) in the treatment of heart failure, we first examined the expression of its target, β3 integrin, in human non‐failing heart, non‐failing hypertrophic heart, and dilated and ischaemic cardiomyopathies. Western blotting for β3‐integrin expression in human left ventricular biopsy lysates from separate patients indicated that although there were no differences in the β3‐integrin levels between non‐failing and non‐failing hypertrophic hearts, significantly elevated levels were observed in both human dilated and ischaemic cardiomyopathies when compared with non‐failing heart tissue (Figure 1A). Immunostaining for β3 integrin showed that β3 integrin was present in human non‐failing, dilated, and ischaemic hearts but less so in non‐failing hearts with hypertrophy, corroborating the western blotting results (Figure 1B). Taken together, these data suggest that β3 integrin is present in human heart failure tissue and thus could provide a possible target for treatment. The pathological features of the human tissue were identified by H&E and Masson's Trichrome staining of the FFPE sections (supplementary material, Figure S1).

### Experimental heart failure in mice

Abdominal aortic constriction (AAC) surgery in mice recapitulates the salient features of human heart failure including pressure overload‐induced left ventricular cardiac hypertrophy and fibrosis 30, 31 and provides a model to test new therapeutic strategies for the treatment of this condition. Previous work has shown that treatment with ldCil is sufficient to enhance pathological angiogenesis in cancer models 27. Thus, we hypothesised that ldCil treatment may enhance cardiac angiogenesis and blood flow after AAC surgery, and thereby provide a strategy to correct the pathobiology of heart failure.

**Figure 2:**
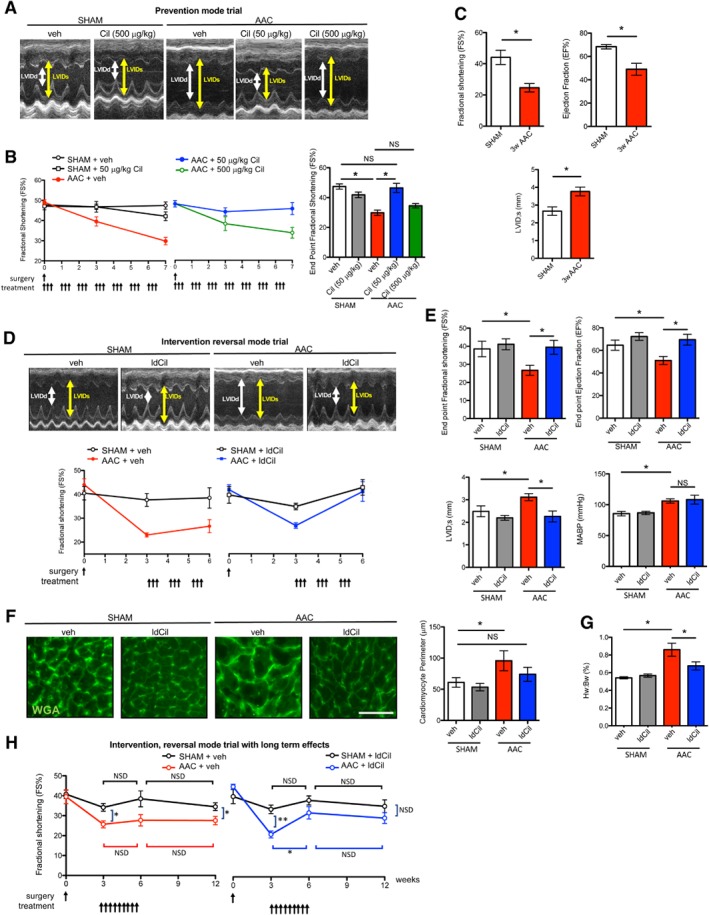
Low‐dose cilengitide treatment restores the effect of pressure overload induced by abdominal aortic constriction. (A) Wild‐type mice underwent either sham or abdominal aortic constriction (AAC) surgery and were treated with either cilengitide (Cil, 50 or 500 μg/kg) or vehicle alone immediately after surgery in a preventative mode trial. Representative endpoint echocardiographic images in vehicle and Cil‐treated sham‐surgery or AAC‐surgery mice (*n*, seven mice per group). Double‐headed arrows, LV internal diameter at diastole (LVIDd) and systole (LVIDs). (B) Treatment of mice with 50 μg/kg, but not 500 μg/kg, cilengitide corrected the fractional shortening effects after AAC surgery. Line graphs show summary fractional shortening (FS %) analysis over time, and the bar chart the endpoint FS % from the mice treated in A. (C) Three weeks after AAC surgery, both FS % and the ejection fraction (EF %) were reduced, whilst left ventricular internal diameter (during systole) (LVID;s) was enhanced compared with sham controls (*n*, five mice per group). (D) Treatment of mice with ldCil rescued the fractional shortening effects of AAC surgery in an intervention mode trial. Wild‐type mice underwent either sham or AAC surgery and 3 weeks after surgery, were treated with either vehicle alone or 50 μg/kg Cil (low‐dose cilengitide, ldCil) three times a week for 3 weeks. Representative endpoint echocardiographic images in sham‐surgery or AAC‐surgery mice treated with either vehicle or ldCil from week 3 to week 6 post‐surgery (*n*, seven mice per group). Line graphs, summary fractional shortening (FS %) over time. (E) Functional parameters from mice in D: endpoint FS %, EF %, and LVID;s were rescued after ldCil treatment in mice after AAC surgery. Mean arterial blood pressure (MABP) was not affected by ldCil treatment (*n*, three mice per group). (F) Sections of myocardium from mice in D stained with fluorescence‐labelled wheat germ agglutinin (WGA) to detect cell perimeters. Image analysis of cardiomyocyte perimeter indicates that treatment with ldCil was sufficient to rescue the myocyte size defect compared with mice with sham surgery (*n*, three mice per group). (G) Heart weight normalised to body weight across treatment groups (*n*, seven mice per group). (H) Treatment with ldCil provided sustained recovery after AAC surgery. Wild‐type mice underwent either sham or AAC surgery. From 3 to 6 weeks, post‐surgery mice were either treated with ldCil or given vehicle alone as control, and fractional shortening was measured at 0, 3, 6, and 12 weeks post‐surgery. Line graphs, summary FS % over time. At 3 weeks post‐AAC surgery, FS % was significantly reduced in mice after AAC surgery and this effect did not recover in mice given vehicle alone. In contrast, mice treated with ldCil from 3 to 6 weeks post‐AAC surgery showed recovery in FS % that was sustained for 6 weeks after treatment cessation (*n*, three to six mice per group). Mean ± SEM. Statistical analysis in B–G: one‐way ANOVA with Tukey's *post hoc* analysis. Statistical analysis in H: non‐parametric test Mann–Whitney. *p < 0.05' **p < 0.01. NS, not significant. Scale bar in F = 20 μm.

Cardiac function was measured by fractional shortening (FS %) first in a prevention mode experimental trial. Cilengitide, or vehicle alone as a control, was administered to mice three times a week for 7 weeks immediately after sham or AAC surgery. At the experimental endpoint (7 weeks post‐surgery), mice that had undergone AAC surgery and had been treated with vehicle alone showed significantly reduced fractional shortening compared with mice that had had sham surgery with vehicle treatment. Administration of ldCil (50 μg/kg) after sham surgery also had no significant effect on fractional shortening. In contrast, treatment of mice with 50 μg/kg ldCil, but not a higher dose of 500 μg/kg, rescued the effect of AAC surgery and restored fractional shortening to that observed in sham‐treated mice (Figure 2A,B). These data suggested that treatment of mice immediately after AAC surgery with ldCil, at a dose similar to that which enhanced pathological angiogenesis in cancer models 27, was sufficient to rescue adverse heart failure effects in fractional shortening.

We tested the effect of ldCil in a more clinically‐relevant intervention reversal study starting treatment 3 weeks after AAC surgery, i.e. after heart failure symptoms were established. Three weeks post‐AAC surgery, FS % and the ejection fraction (EF %) were significantly reduced, while left ventricle systole (LVID) was increased, compared with mice that had undergone sham surgery (Figure 2C). To test the effect of ldCil treatment, mice underwent either sham or AAC surgery and 3 weeks later were treated with either vehicle or ldCil three times a week for 3 weeks. At the end of treatment, although vehicle treatment had no apparent modifying effects, ldCil treatment rescued the effects on endpoint FS %, EF %, and LVID (Figure 2D,E). Importantly, these effects were independent of changes in mean arterial blood pressure (MABP) (Figure 2E). In addition to these functional features, mice that have undergone AAC surgery typically exhibit adverse effects of enhanced cardiac hypertrophy with concomitant increased heart‐to‐body weight ratios 30, 32. Wheat germ agglutinin (WGA) binds to cell membrane glycoproteins and is used to determine the cross‐sectional sizes of myocytes as a measure of cardiomyocyte hypertrophy 33. Cardiomyocyte perimeter measurements in WGA‐stained sections of heart from experimental endpoints were reduced in ldCil‐treated mice after AAC surgery, compared with vehicle (Figure 2F). Further, ldCil treatment blunted increases in heart‐to‐body weight ratios in mice after AAC surgery (Figure 2G). Thus, in this intervention mode study, ldCil treatment restored cardiac function and structure in the AAC model of heart failure to levels observed with sham surgery.

To test whether ldCil treatment efficacy was maintained, even after treatment cessation, mice were given ldCil or vehicle from weeks 3 to 6 post‐sham or AAC surgery and the effects on cardiac contractility were assessed up to 12 weeks post‐surgery. The results indicated that FS % was not significantly altered between 3 and 6 weeks or between 6 and 12 weeks post‐sham surgery in either vehicle alone or ldCil‐treated mice. As expected, fractional shortening was significantly reduced in vehicle‐treated mice after AAC surgery. This reduction in FS % was sustained up until the 12‐week endpoint, indicating the persistent long‐term effects of AAC surgery in mice receiving vehicle alone. In contrast, treatment with ldCil elevated fractional shortening significantly between 3 and 6 weeks after AAC surgery. This phenotypic rescue was sustained up until 12 weeks post‐surgery, at which point there was no significant difference between ldCil treatment of mice that had undergone sham or AAC surgery (Figure 2H). Thus, intervention treatment with ldCil is sustained after cessation of treatment, is disease‐modifying, and potentially provides an advantage over current treatment strategies.

### Treatment with low‐dose cilengitide enhances cardiac angiogenesis *in vivo*

**Figure 3:**
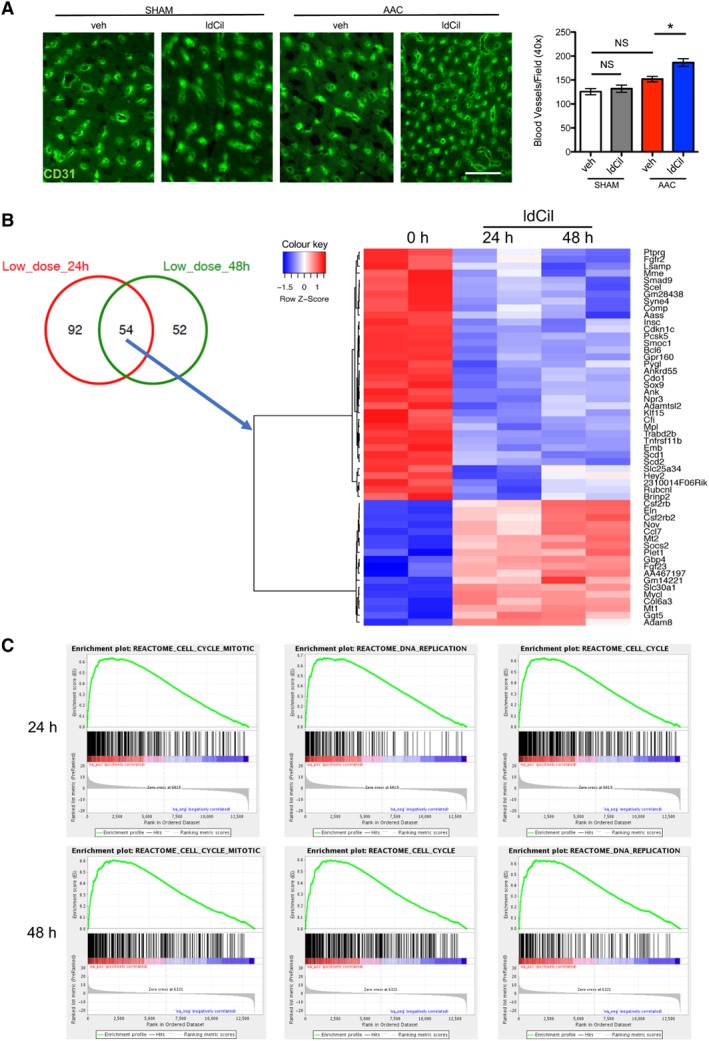
Treatment with low‐dose cilengitide enhances cardiac blood vessel density *in vivo* and stimulates cardiac endothelial cell DNA‐replication, mitosis, and cell‐cycle transition transcriptional profiles. (A) Wild‐type mice underwent either sham or AAC surgery and were treated with ldCil or vehicle control for 3 weeks. Representative immunofluorescence images of myocardium stained for the endothelial cell marker CD31. Treatment with ldCil enhanced blood vessel density (BVD) in the myocardium of mice after AAC but not sham surgery. Mean ± SEM. One‐way ANOVA with Tukey's *post hoc* test; *p < 0.05. NS, not significant. (B) Duplicate preparations of mouse cardiac endothelial cells were treated for 0, 24 or 48 h with ldCil and RNA‐Seq was performed. Heatmap of differentially expressed (DE) up‐ and down‐regulated transcripts in cardiac endothelial cells treated with ldCil. Results are shown for duplicate samples. Fifty‐four DE genes were concordant at 24 and 48 h ldCil treatment compared with control. (C) Enrichment analysis of RNA‐Seq data demonstrates that treatment of cardiac endothelial cells significantly enriches for cell cycle, DNA replication, cell cycle, mitosis, and DNA strand elongation pathways – all involved in cell proliferation, a key process in angiogenesis.

We examined the physiological and molecular changes associated with the effect of ldCil in the AAC model. Since heart failure is associated with reduced blood flow to the heart, and we have published previously that ldCil can increase blood vessel density in pathological angiogenesis 27, we examined cardiac capillary density after AAC surgery. At 2, 3, and 6 weeks post‐AAC surgery, heart sections were immunostained for CD31 antibody to detect blood vessels, and the number of blood vessels per field of view was assessed. Capillary density was not affected in the heart after AAC (supplementary material, Figure S2). In contrast, ldCil treatment significantly enhanced cardiac blood vessel density in mice after AAC surgery but had no significant effect in mice that had undergone sham surgery (Figure 3A). Interestingly, this dose of 50 μg/kg cilengitide was the same dose that enhanced tumour angiogenesis in previous studies 27, 28. The data indicate that the elevated cardiac function induced by treatment with ldCil after AAC surgery was associated with enhanced cardiac blood vessel density. RNA‐Seq analysis, to define transcriptomic profiles of mouse cardiac endothelial cells treated with ldCil for 24 and 48 h, identified 54 concordantly differentially‐expressed genes following 24 and 48 h of ldCil treatment (Figure 3B and supplementary material, Figure S3). Indeed, common differentially‐upregulated genes at both 24 h and 48 h of ldCil treatment included several known or putative pro‐angiogenic or cardioprotective regulators such as *Csf2rb* 34, *Eln* 35, *Nov* (encoding a known ligand of αvβ3 integrin 36), *Ccl7* (*CCL7* known to improve cardiac repair 37), *Mt2* 38 *Socs2* 39 *Fgf23* (known to protect against cardiac dysfunction 40), *Slc30a1* 41 *Col6a3* 42 *Mt1* 43, *Ggt5* 44 and *Adam8* 45. Furthermore, enrichment analysis of RNA‐Seq data, at both time points, showed significant increases in the gene sets involved in mitosis, DNA repair, and cell cycle – all features of enhanced angiogenesis and the myocardial response to injury/pressure overload (Figure 3C).

### ldCil treatment restores the transcriptomic profiles in the AngII‐stimulated model of cardiac hypertrophy similar to those found in non‐failing human heart

**Figure 4:**
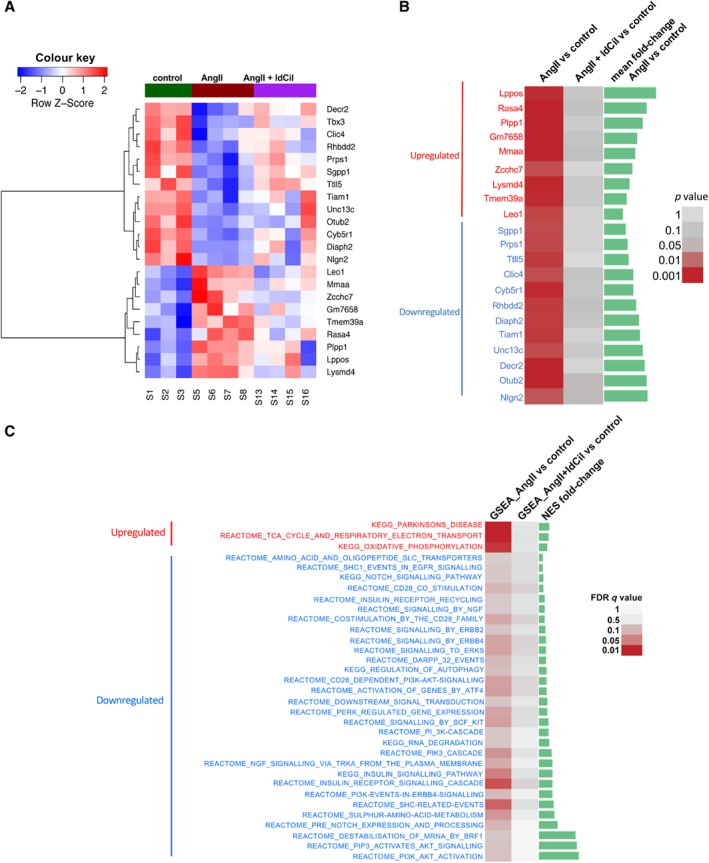
Low‐dose cilengitide treatment reverses the transcriptomic profiles of AngII‐stimulated cardiomyocytes to control levels. (A) Mouse cardiomyocytes were isolated and either exposed to angiotensin II (AngII) to mimic the molecular stress of heart failure or exposed to AngII and also treated with ldCil. Heatmap of differentially expressed protein coding genes indicates that AngII stimulation resulted in 13 down‐ and nine up‐regulated transcripts. ldCil treatment of AngII‐stimulated cardiomyocytes rescues the differentially expressed (DE) transcript profiles. (B) Data from A represented in order of fold‐change. DE protein‐coding genes with **p < 0.01 in AngII versus control, also with high average expression across samples, became not DE (*p* > 0.05) in the AngII + ldCil group compared with control. Green bars show the levels of fold‐change. (C) Treatment with ldCil restores aberrant signalling pathways induced by AngII stimulation of cardiomyocytes. GSEA of KEGG and reactome signalling pathways reveal that dysregulated pathways with FDR *q* < 0.1 in AngII versus control became not significant (*q* > 0.1) in AngII + ldCil compared with control.

Previous studies have identified that angiotensin II (AngII) treatment of cardiomyocytes mimics many features of hypertrophy observed in heart failure *in vivo* 46. The direct effect of ldCil on cardiomyocytes was examined: AngII‐stimulated cardiomyocytes in culture exhibited a significant increase in cell size, supporting the previously published effects on hypertrophy and corroborating the use of AngII stimulation of cardiomyocytes as a model for hypertrophy (supplementary material, Figure S4) 46. Another key feature of hypertrophy observed in both human heart failure and AngII‐treated mouse cardiomyocytes is the enrichment of foetal‐like gene programmes involved in cardiac remodelling 47, including upregulation of hypertrophic markers such as *Myh7*. Importantly, additional treatment with ldCil restored *Myh7* levels back to untreated levels (supplementary material, Figure S5). These data not only validated the utility of AngII stimulation of cardiomyocytes as a model for hypertrophy but also indicated that ldCil has a direct effect on cardiomyocytes to restore this feature of hypertrophy. Additionally, RNA‐Seq transcriptome analysis demonstrated that AngII stimulation of cardiomyocytes resulted in significant differential expression changes in multiple genes (Figure 4A,B). Some of the most significantly differentially expressed genes included the upregulation of LIM domain containing preferred translocation partner (*Lppos*), the haploinsufficiency of which has been reported to be involved in developmental heart defects 48; *Rasa4*, RAS p21 protein activator1, which has been shown to regulate cardiac fibroblast activation, although no known function in cardiomyocytes has been reported to date 49; and the downregulation of neuroglin 2 (*Nlgn2*) polymorphisms, which have been shown to be associated with blood pressure changes 50; whilst *Otub2*, which encodes the ovarian tumour domain (OTU)‐containing subfamily of deubiquitinating enzymes, has been associated with multi‐systemic human disorders including heart failure 51. Treatment with ldCil restored the statistically significant transcriptomic alterations induced by AngII back to control treated cardiomyocyte levels for all the candidates shown (Figure 4A,B). Gene sequence enrichment analysis (GSEA) showed that AngII stimulation induced significant differential expression of multiple pathways (FDR *q* < 0.1). Once again, after additional treatment with ldCil, these changes were no longer significant (FDR *q* > 0.1) (Figure 4C), suggesting that treatment with ldCil rescues the signalling pathway alterations induced by AngII. In particular, the significant increase in TCA and oxidative stress pathways associated with AngII exposure and associated with some forms of heart failure 52 are restored by treatment with ldCil. Additionally, PI3K and Akt signalling pathways are the most significantly downregulated after AngII exposure and are known to be involved in heart failure responses 53. Indeed, both of these pathways are highly expressed after treatment with ldCil (Figure 4C). Taken together, these data further corroborated the notion that the direct effects of ldCil on cardiomyocytes could be involved in restoring the pathobiology and molecular effects of hypertrophy.

### ldCil treatment restores transcriptomic changes associated with non‐failure in human heart

**Figure 5:**
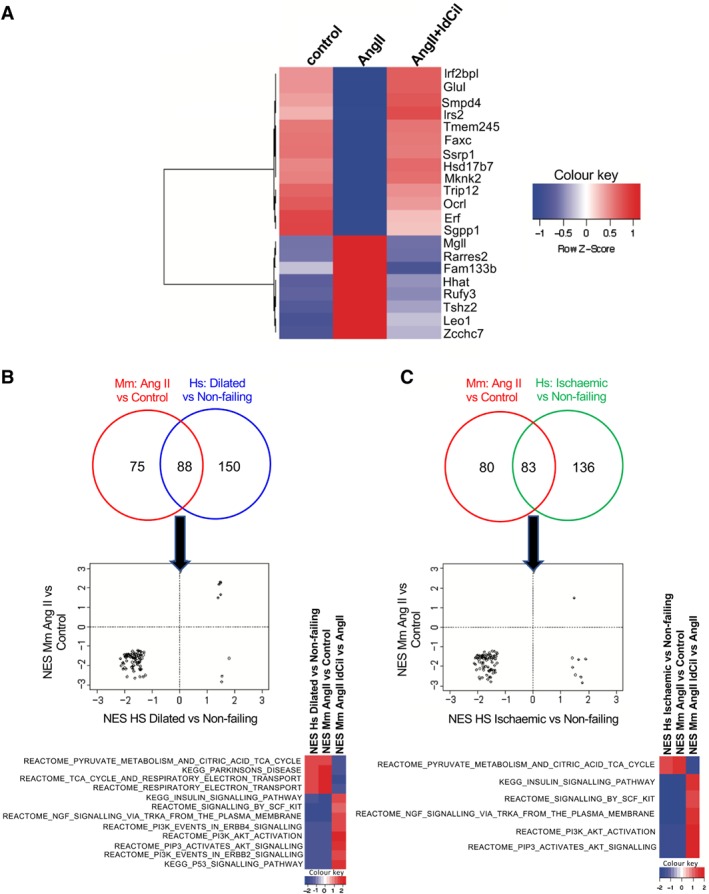
Low‐dose cilengitide treatment restores transcriptomic profiles in mouse cardiomyocytes similar to those found in non‐failing human heart. (A) Treatment of mouse cardiomyocytes with AngII + ldCil compared with AngII restores the transcriptomic signature similar to that found in human non‐failing versus failing heart. Heatmap illustrates concordant DE transcripts between human failing (ischaemic and dilated cardiomyopathies) versus non‐failing heart and mouse cardiomyocytes treated with AngII versus control or AngII versus AngII + ldCil. (B) Venn diagrams show the overlap of gene sequence expression analysis (GSEA) pathways (*p* < 0.05) in mouse cardiomyocytes stimulated with AngII versus control and human dilated cardiomyopathy (CMP) versus non‐failing heart. Scatterplots of normalised enrichment scores (NES) of the overlapping pathways illustrate how many of those change concordantly. Heatmap of NES for GSEA pathways that change concordantly in human dilated CMP versus non‐failing heart, mouse AngII‐stimulated versus control cardiomyocytes, and AngII‐stimulated cardiomyocytes treated with ldCil versus AngII‐stimulated cardiomyocytes. (C) Venn diagrams of the overlap of GSEA pathways (*p* < 0.05) in mouse cardiomyocytes stimulated with AngII versus mouse control and human ischaemic CMP versus non‐failing heart. Scatterplots of NES of the overlapping pathways illustrate how many of those change concordantly. Heatmap of NES for GSEA pathways that change concordantly in human ischaemic versus human non‐failing heart, mouse AngII‐stimulated versus control mouse cardiomyocytes, and AngII‐stimulated mouse cardiomyocytes treated with ldCil versus AngII‐stimulated mouse cardiomyocytes.

Previous work, using RNA isolated from bulk human cardiac samples, identified the transcriptomic changes that are significantly regulated in human heart failure 54. To examine the relevance of our findings in mouse tissue to human heart disease, we identified concordant transcriptomic changes published for human failing heart (ischaemic and/or dilated versus human non‐failing) versus those in the mouse model of hypertrophy (AngII‐stimulated cardiomyocytes versus control treated cardiomyocytes). We found 290 orthologous genes altered concordantly in mouse cardiomyocytes and human failing versus non‐failing heart. A subgroup of these genes (13 downregulated and 8 upregulated) were restored to non‐failing human heart levels after treatment of mice with ldCil (Figure 5A). Furthermore, out of the 163 differentially regulated pathways in AngII‐stimulated mouse cardiomyocytes versus control cardiomyocytes, 88 were common with the 238 differentially enriched pathways in human dilated heart versus non‐failing heart (Figure 5B and supplementary material, Figure S6A). Gene sequence enrichment analyses revealed that four concordantly upregulated and eight concordantly downregulated pathways in human idiopathic dilated cardiomyopathy versus non‐failing heart and mouse AngII‐stimulated cardiomyocytes versus control were restored to non‐failing human heart profiles after ldCil treatment (Figure 5B, heatmap and supplementary material, Figure S6A). Similarly, out of the 163 differentially regulated pathways in AngII‐stimulated mouse cardiomyocytes versus control cardiomyocytes, 83 were common with the 219 differentially enriched pathways in human ischaemic heart versus non‐failing heart (Figure 5C and supplementary material, Figure S6B). GSEA uncovered that six concordant pathways, one upregulated and five downregulated, in human ischaemic heart versus non‐failing heart and mouse AngII‐stimulated cardiomyocytes versus control, were restored after treatment with ldCil (Figure 5C, heatmap and supplementary material, Figure S6B). Additionally, some of these concordantly enriched pathways were similar for both idiopathic dilated and ischaemic cardiomyopathy, including insulin signalling pathways, SCF–Kit signalling, NGF signalling, PI3K signalling, and AKT signalling, suggesting common mechanistic signatures that are relevant to the recovery of both cardiomyopathies. Western blotting analyses validated some of the differentially expressed TCA‐cycle enzymes at the protein level (supplementary material, Figure S7). Taken together, these data provide evidence that treatment of AngII‐stimulated cardiomyocytes with ldCil generates molecular profiles that are associated with human non‐failing heart.

## Discussion

Heart failure is a common feature of the final stages of several cardiovascular diseases [e.g. hypertension, myocardial infarction (MI)], with characteristic pathologies of reduced microvascular blood flow and cardiomyocyte hypertrophy. Importantly, disease‐modifying strategies with longer‐lasting pharmacodynamics are required.

Some studies have implicated β3 integrin as a mediator of cardiac remodelling, suggesting that signalling downstream of this integrin could be disease‐modifying 17, 20, 21, 55. Heart failure is associated with poor blood vessel perfusion and thus increasing perfusion could be beneficial. Cilengitide is an RGD‐mimetic that targets αvβ3 integrin 56, 57, 58. In cancer studies, low, but not high, doses of this agent induce vascular promotion in pathological conditions, and can also enhance chemosensitivity in malignant cells 27, 28.

We hypothesised that because ldCil can have pro‐angiogenic effects in cancer, it may also have vascular‐promoting effects in heart failure models that could exert beneficial disease‐modifying actions. Here, we have shown that treatment with ldCil enhances cardiac angiogenesis in the AAC model of heart failure and inhibits pathological hypertrophy of cardiomyocytes. Moreover, ldCil treatment recapitulates some of the transcriptomic profiles of human non‐failing hearts, suggesting that this strategy may have relevance to human heart failure treatment. Furthermore, we have shown that β3‐integrin expression is enhanced in human dilated and ischaemic cardiomyopathies.

Current treatments for heart failure are not disease‐modifying when treatment ceases and thus their efficacy relies on treatment for life 59. It is of interest that the effects of ldCil in the AAC model of heart failure show potentially long‐lasting effects. The reasons why this vascular promotion effect inducing a pro‐angiogenic transcriptomic profile may be effective compared with previous pro‐angiogenic strategies such as protein tyrosine phosphatase 1B (PTP1B) inactivation 60, 61 or vascular endothelial growth factor‐induced neovascularisation 62 are not known currently. One possibility is that the endothelial cells lining blood vessels not only act as conduits for blood flow but also produce their own set of paracrine factors, namely angiocrine factors, which can have effects on development and the repair of surrounding tissue 63. This is a relatively new, but exciting, concept in understanding the role of cardiac angiocrine factors in cardiac repair 64. Recognising that angiocrine biology has been related to integrin‐mediated mechanosensing 65 or signalling downstream of endothelial cell integrins 66 it is possible that angiocrine factor production after ldCil treatment may be of significance. Although beyond the scope of the current study, it would be of interest to investigate whether the ldCil‐treatment‐induced paracrine factors, identified in this study, also aid in cardiac tissue repair. We also speculate that because ldCil treatment has direct effects on cardiomyocyte function and hypertrophy, as well as its pro‐angiogenic effects, the combination of these effects on at least two cell types in the heart would provide improved restoration of the pathophysiological features of heart failure.

Our *in vitro* data describe how the effects of ldCil on AngII‐stressed cardiomyocytes correlate with the published transcriptomic signature of a human normal heart compared with failing heart 54. The common transcriptomic signature suggests that if the *in vivo* ldCil effects on cardiomyocytes are similar to those we discovered *in vitro*, then ldCil treatment could have a restorative effect on cardiomyocyte pathophysiology that may be relevant to human heart disease. Limitations of our study include the relatively few, but statistically relevant, mice used in the *in vivo* AAC experiments, together with the fact that AAC is a model of heart failure that does not include some of the pathophysiological causes of the disease in humans, such as lack of exercise or smoking. Additionally, the expected high mortality rates of mice at 12 weeks post‐AAC surgery limited the design of the longer‐term experiments presented. Despite these limitations, our data do correlate human heart failure with our observations in mouse models, providing some confidence in the interpretation of the results. Overall, our results suggest that treatment with ldCil merits further investigation for disease‐modifying effects in heart failure.

## Author contributions statement

MD carried out the majority of the experiments with help from LR and AA. RSB helped to train MD in the early stages of the project. GD'A assisted with cell preparations and analysis. EM and JW carried out the bioinformatics analyses for both mouse and human data. OC and VS‐M performed Masson's Trichrome staining. KCB and KBM provided the human cardiac tissue samples. KHD wrote the first draft of the manuscript and all the authors contributed to the manuscript revisions. KHD and AH conceived, designed, and supervised the study.

## Supporting information


**Supplementary materials and methods**
**Figure S1.** Histological changes in human non‐failing heart, non‐failing hypertrophic heart, and ischaemic and dilated cardiomyopathies
**Figure S2.** AAC surgery does not affect cardiac blood vessel density
**Figure S3.** Transcriptomic changes in cardiac endothelial cells after treatment with low‐dose cilengitide
**Figure S4.** Angiotensin II treatment stimulates cardiomyocyte enlargement *in vitro*
**Figure S5.** ldCil treatment after AAC surgery restores *Myh7* transcript levels
**Figure S6.** KEGG and REACTOME data indicate overlap between ldCil‐treated AngII‐stimulated cardiomyocytes and non‐failing human heart enriched transcriptional pathways
**Figure S7.** Protein validation of common differentially expressed TCA‐cycle enzymes in non‐failing human and ldCil‐treated AngII‐stressed cardiomyocytes
