# Supplementary material for: Repurposing an anti‐cancer agent for the treatment of hypertrophic heart disease
Source: J Pathol. 2019 Oct 30;249(4):523–35. doi: 10.1002/path.5340 (PMC6900130; doi:10.1002/path.5340)
Supplement: Supplementary file 1 — Supplementary materials and methods Figure S1. Histological changes in human non‐failing heart, non‐failing hypertrophic heart, and ischaemic and dilated cardiomyopathies Figure S2. AAC surgery does not affect cardiac blood vessel density Figure S3. Transcriptomic changes in cardiac endothelial cells after treatment with low‐dose cilengitide Figure S4. Angiotensin II treatment stimulates cardiomyocyte enlargement in vitro Figure S5. ldCil treatment after AAC surgery restores Myh7 transcript levels Figure S6. KEGG and REACTOME data indicate overlap between ldCil‐treated AngII‐stimulated cardiomyocytes and non‐failing human heart enriched transcriptional pathways Figure S7. Protein validation of common differentially expressed TCA‐cycle enzymes in non‐failing human and ldCil‐treated AngII‐stressed cardiomyocytes [file PATH-249-523-s001.docx]

**Repurposing an anti-cancer agent for the treatment of hypertrophic heart disease**

Dukinfield M *et al. J Pathol* DOI: 10.1002/path.**5340**

**Supplementary Figures S1–S7**

**
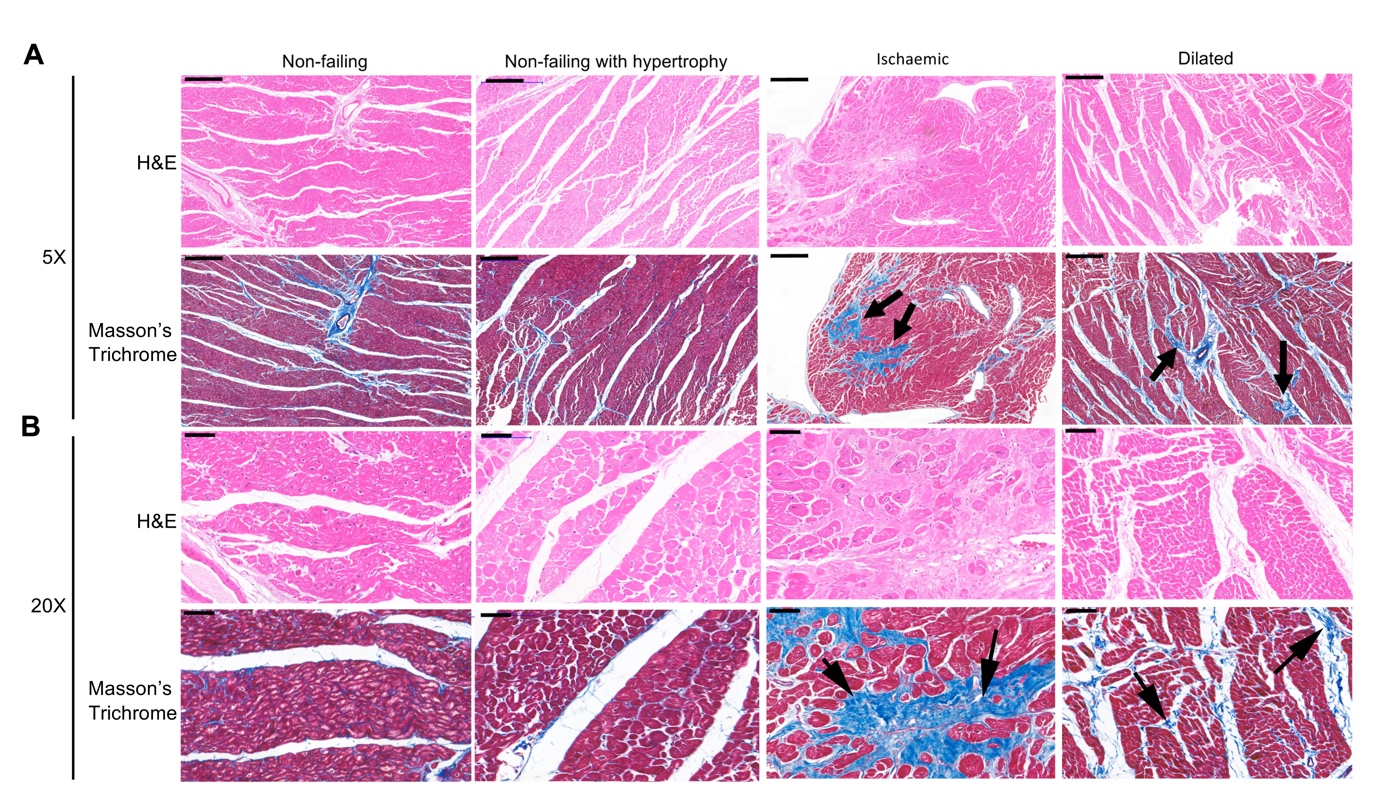
**

**Figure S1. Histological changes in human non-failing heart, non-failing hypertrophic heart, and ischaemic and dilated cardiomyopathies.** Representative images of H&E- and Masson’s Trichrome-stained sections of non-failing heart, non-failing heart with hypertrophy, and ischaemic and dilated cardiomyopathy. Non-failing heart with hypertrophy shows an increase in myocyte size. Ischaemic and dilated cardiopathies show elevated fibrotic responses. Black arrows, fibrosis. Scale bars: (A) 5× objective, 500 μm; (B) 20× objective,100 μm.

**
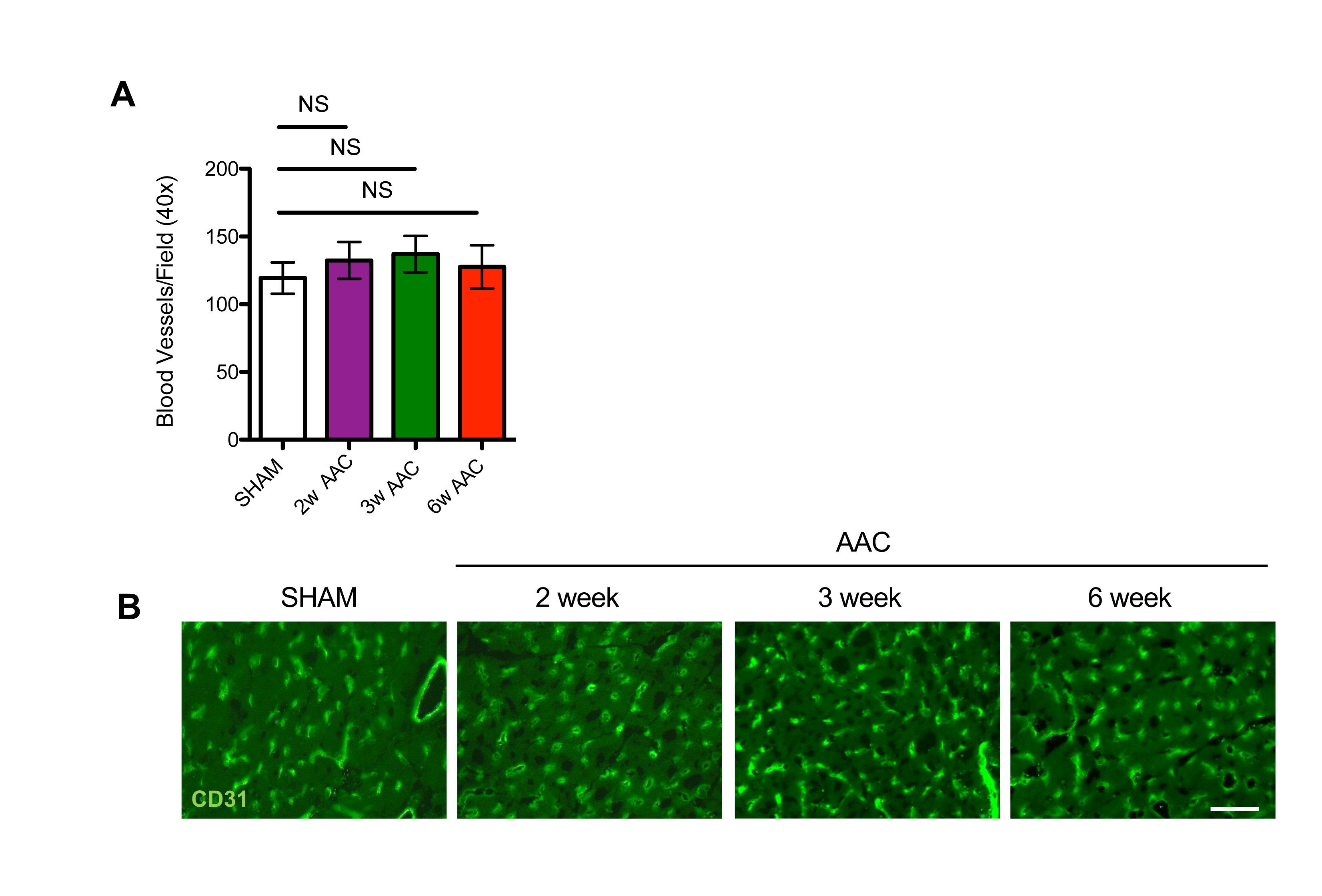
**

**Figure S2.** **AAC surgery does not affect cardiac blood vessel density.** Wild-type mice underwent either sham or AAC surgery. (A) Bar chart represents numbers of CD31-positive blood vessels/high-power field of view (40× objective) of hearts from mice that underwent sham surgery or 2, 3, and 6 weeks post-AAC surgery. (B) Representative images of CD31-immunofluorescence in mouse myocardium. Mean ± SEM. Statistical analysis by one-way ANOVA with Tukey’s multiple comparison *post hoc*. NS, not significant. Scale bar = 20 μm.


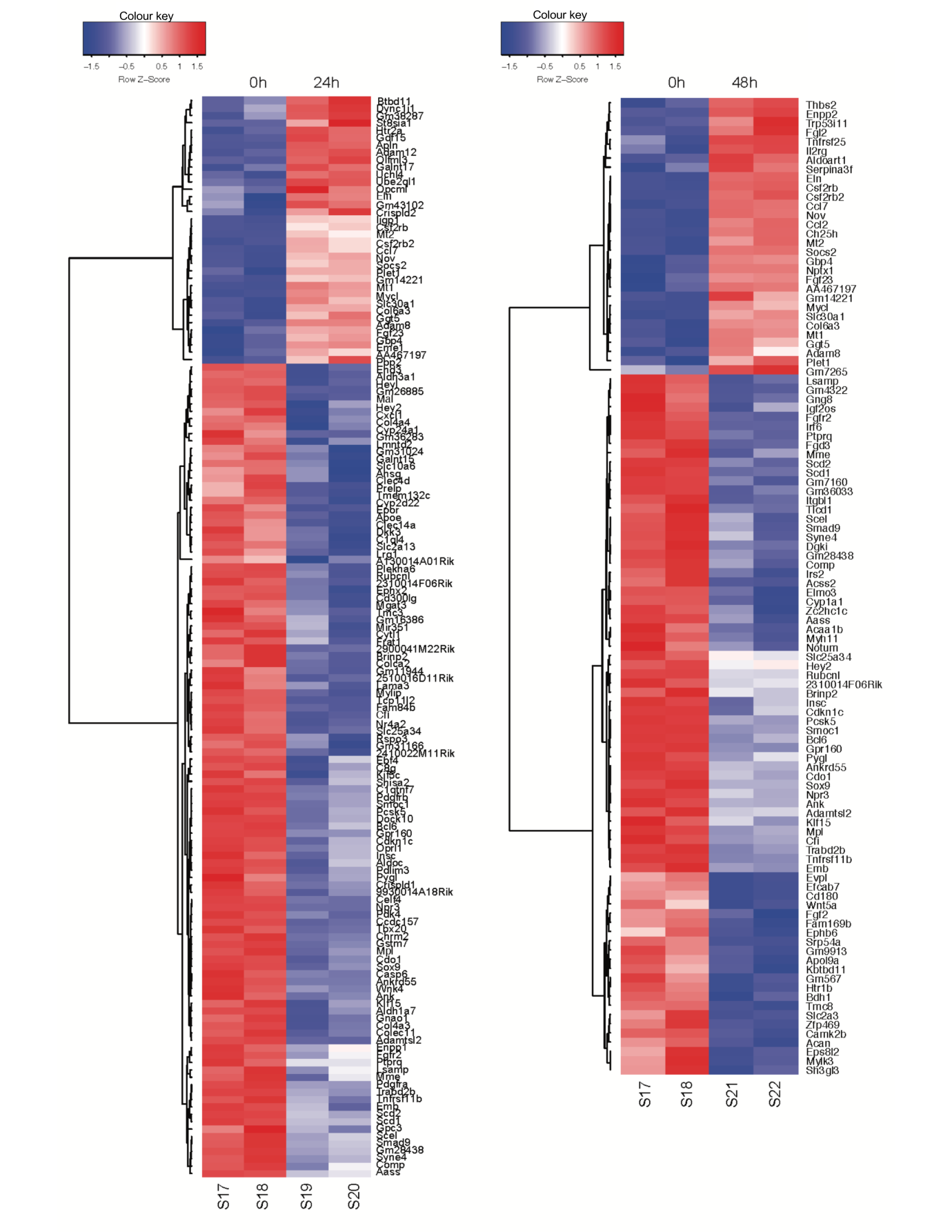


**Figure S3.** **Transcriptomic changes in cardiac endothelial cells after treatment with low-dose cilengitide.** Cardiac endothelial cells were isolated and treated with vehicle alone (control) for 24 h or 48 h with low-dose cilengitide (ldCil, 20 nm). Two separate samples were prepared for each condition. (A) At 24 h of 20 nm cilengitide treatment, 146 genes were differentially expressed (DE; FDR *q* < 0.05 and absolute log_2_ FC > 1). (B) At 48 h of 20 nm cilengitide treatment, 107 genes were DE (FDR *q* < 0.05 and absolute log_2_ FC > 1).


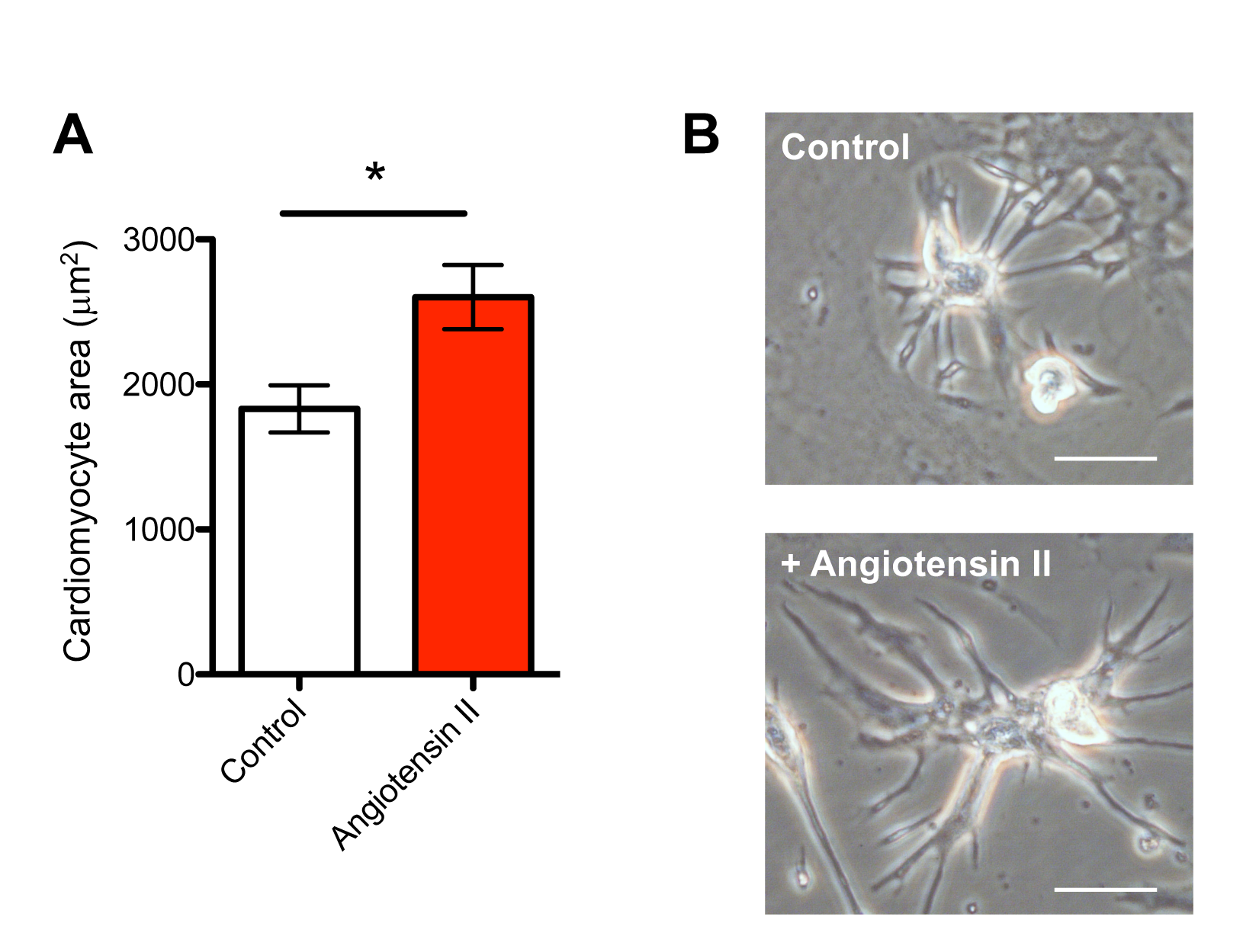


**Figure S4.** **Angiotensin II treatment stimulates cardiomyocyte enlargement *in vitro*.** Cardiomyocytes were cultured *in vitro* and treated with vehicle alone or AngII. (A) Bar chart indicates areas of control and AngII-treated cardiomyocytes. Mean ± SEM. Statistical analysis by Student’s *t*-test; **p* < 0.05 versus control. (B) Representative images of single cardiomyocytes. Scale bar = 20 μm.

**
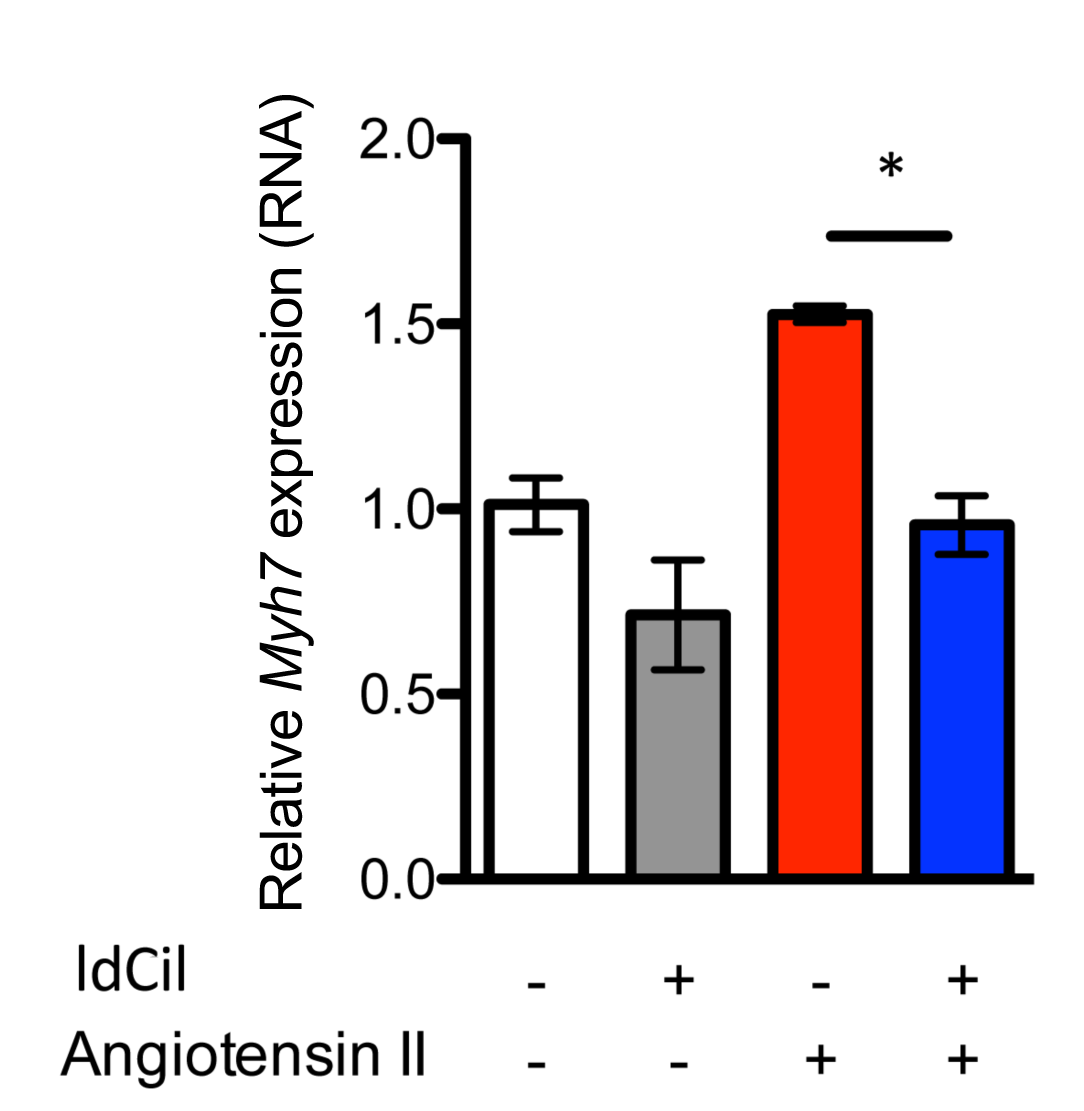
**

**Figure S5.** **ldCil treatment after AAC surgery restores *Myh7* transcript levels.** Mice underwent either sham or AAC surgery and were treated with vehicle alone or ldCil 3 weeks after surgery. Bar chart shows relative *Myh7* transcript expression from whole heart preparations, normalised to *Gapdh*. Mean ± SEM. Statistical analysis by one-way ANOVA with Tukey’s multiple comparison test *post hoc*. **p* < 0.05; *n* = 6 hearts per group.

**Figure S6. KEGG and REACTOME data indicate overlap between ldCil-treated AngII-stimulated cardiomyocytes and non-failing human heart enriched transcriptional pathways.** (A) Whole GSEA analyses underlying the data given in the heatmap of Figure 4E. Twenty-five common pathways were found when comparing GSEA data from mouse AngII-stimulated cardiomyocytes versus control cardiomyocytes; AngII-stimulated versus AngII-stimulated plus ldCil treatment and human idiopathic cardiac myopathy versus non-failing heart. Of these 25 pathways, 12 changed concordantly: four concordantly upregulated and eight downregulated pathways. (B) Whole GSEA analyses underlying the data given in the heatmap of Figure 4F. Eighteen common pathways were found when comparing GSEA data from mouse AngII-stimulated cardiomyocytes versus control cardiomyocytes; AngII-stimulated versus AngII-stimulated plus ldCil treatment and human ischaemic cardiac myopathy versus non-failing heart. Of these 18 pathways, six changed concordantly. Taken together, these data support that ldCil treatment reverses molecular signatures that are associated with human failing heart back to that of human non-failing heart.

***
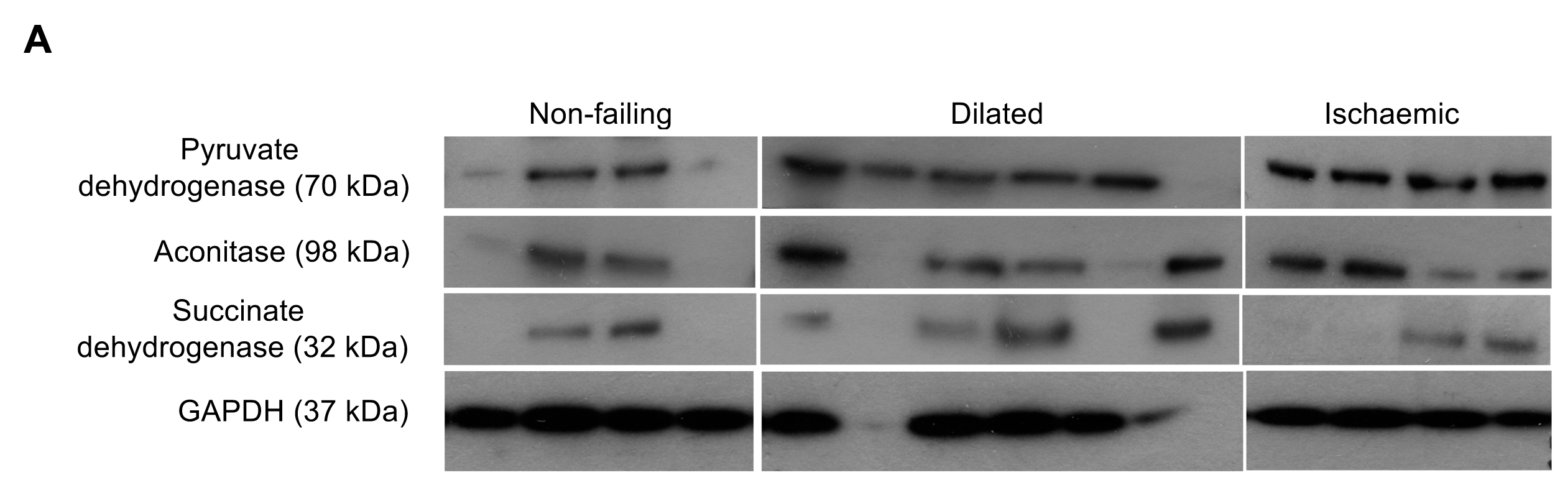
***


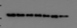

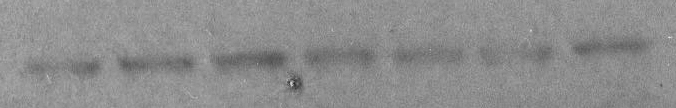


Ctrl

AngII

AngII + ldCil

Pyruvate

dehydrogenase

(70 kDa)

GAPDH

(37 kDa)

**B**

Ctrl

AngII

AngII +

ldCil

**Figure S7. Protein validation of common differentially expressed TCA-cycle enzymes** **in non-failing human and ldCil-treated AngII-stressed cardiomyocytes.** Western blotting and densitometric analysis. (A) Protein validation of RNA-Seq data hits from Figure 5E,F showing upregulation of the TCA-cycle enzymes aconitase, pyruvate dehydrogenase, and succinate dehydrogenase in dilated and ischaemic human cardiomyopathy whole-tissue protein lysates versus non-failing human heart protein lysates. *n*, 4–6 human tissue sample lysates per tissue type. Mean ± SEM. Student’s *t*-test, **p* < 0.05; ***p* < 0.01. (B) Low-dose cilengitide treatment of AngII-stressed mouse cardiomyocytes rescues the elevated expression of the TCA-cycle enzyme pyruvate dehydrogenase found to be upregulated in human failing hearts. Mean ± SD. *N*, 2 or 3 mouse cardiomyocyte preparations. Student’s *t*-test, **p* < 0.05.
